# Rapid on–off effects of methotrexate on lymphocytes may facilitate recovery of suppressed vaccine immunogenicity after its short-term methotrexate discontinuation in rheumatoid arthritis

**DOI:** 10.3389/fimmu.2026.1898581

**Published:** 2026-08-07

**Authors:** Heejin Jeong, Seojin Yang, Jun Won Park, Kevin Winthrop, Eun Bong Lee, Jin Kyun Park

**Affiliations:** 1 Department of Molecular Medicine and Biopharmaceutical Sciences, Graduate School of Convergence Science and Technology, Seoul National University, Seoul, Republic of Korea; 2 Division of Rheumatology, Department of Internal Medicine, Seoul National University College of Medicine, Seoul, Republic of Korea; 3 Division of Infectious Diseases, Oregon Health and Science University, Portland, OR, United States

**Keywords:** methotrexate, pharmacokinetics, rheumatoid arthritis, suppression, vaccination

## Abstract

**Objective:**

Short-term methotrexate (MTX) discontinuation restores vaccine responses in rheumatoid arthritis (RA). We investigated how the timing of MTX exposure relative to lymphocyte activation influences cellular pharmacokinetics and vaccine immunogenicity.

**Methods:**

T and B cells from healthy donors (n = 4) were stimulated and treated with MTX either continuously or as a 24-hour pulse at defined activation time points. Activation, proliferation, and apoptosis were assessed by flow cytometry. Intracellular MTX polyglutamate (MTX-PG) levels were quantified using liquid chromatography mass spectrometry. Expression of reduced folate carrier 1 (RFC1), folylpolyglutamate synthase (FPGS), and γ-glutamyl hydrolase (GGH) was quantified. A *post-hoc* analysis of two clinical trials including 318 RA patients evaluated the effects of MTX timing on influenza vaccine responses.

**Results:**

Continuous MTX suppressed activation and proliferation and induced apoptosis in activated, but not resting T and B cells from healthy controls. A 24-hour MTX pulse on days 2 and 3 of activation inhibited cell activation and proliferation. Activated cells accumulated rapidly intracellular MTX-PG, peaking at 24 hours of MTX exposure, followed by near-complete clearance by 48 hours after MTX withdrawal. Resting cells showed minimal MTX-PG accumulation. Protein expression of RFC1 and FPGS peaked at 48 hours post-activation and declined thereafter. In RA patients, MTX administration on days 3–4 post-vaccination impaired vaccine responses, whereas delaying MTX for one week post-vaccination had minimal impact.

**Conclusions:**

MTX has a rapid intracellular turnover and exerts activation-phase-dependent effects on lymphocytes, mediated in part by dynamic expression of RFC1 and FPGS. These might explain the rapid recovery of vaccine responses after brief MTX interruption.

## 1 Introduction

Methotrexate (MTX) suppresses immune response and reduces vaccine efficacy in patients with rheumatoid arthritis (RA) (1–3). A short-term discontinuation of MTX for 1 or 2 weeks after but not before vaccination restored vaccine response to seasonal influenza in patients with RA on chronic MTX therapy (4–6). This relatively rapid restoration of vaccine response following short-term MTX interruption is unexpected since the disease-modifying effects on inflammatory arthritis manifest after 3–6 weeks of MTX treatment. Furthermore, RA disease activity started to flare after 2 weeks of MTX discontinuation (7–9). This discrepancy between the delayed onset of MTX’s disease-modifying effects on joints and its rapid impact on vaccine response suggests that MTX may modulate immune cells more rapidly compared to its disease modifying effects on synovial inflammation.

MTX reaches peak plasma concentration within 1 hour of oral administration and is largely cleared within 24 hours due to its short plasma half-life of approximately 3–4 hours (7, 10). As a structural analogue of folate, MTX has a high affinity for folate-dependent enzymes. It enters cells via folate transporters such as the reduced folate carrier 1 (RFC1) and is subsequently converted into MTX polyglutamates (MTX-PGs) by folylpolyglutamate synthase (FPGS) (11–13). These active metabolites inhibit folate-dependent pathways, suppressing purine and pyrimidine synthesis and can be detected in tissue for weeks (14, 15). In addition, MTX exerts immunomodulatory effects through mechanisms such as adenosine release, nitric oxide synthase uncoupling, and inhibition of Janus kinase-signal transducer and activator of transcription (JAK-STAT) and Nuclear factor kappa B (NF-κB) signaling, ultimately inhibiting cell activation and proliferation (12).

Despite its short plasma half-life of approximately 3–4 hours, MTX is retained intracellularly as MTX polyglutamates (MTX-PGs), which are thought to account for its prolonged pharmacologic effects (12, 16, 17). The prevailing understanding of prolonged intracellular MTX retention is largely based on measurements of MTX-PGs in erythrocytes (20–22). However, little is known about the intracellular pharmacokinetics and pharmacodynamics of MTX in activated lymphocytes.

In RA treatment, MTX is administered once weekly due to its prolonged intracellular retention and sustained effects despite rapid plasma clearance (16, 17). This intermittent “pulse” exposure may differentially affect immune responses depending on the timing of drug exposure relative to critical windows of immune activation, such as the early phase following vaccination, when rapid lymphocyte proliferation and antibody generation occur (18, 19). Therefore, the timing of MTX administration relative to vaccination may be a key determinant of vaccine efficacy.

This study aimed to investigate the activation-dependent effects of MTX on lymphocytes *in vitro* to elucidate mechanisms underlying the rapid restoration of vaccine responses following short-term MTX interruption in patients with RA on chronic MTX treatment.

## 2 Methods

### 2.1 Cell isolation and activation

This study was approved by the Institutional Review Board (IRB) of Seoul National University Hospital (IRB No. 2403-127-1524). Written informed consent was obtained from all participants prior to venous blood collection.

Peripheral blood mononuclear cells (PBMCs) were isolated from healthy donors (**Supplementary Table 1**) using Ficoll-Paque (GE Healthcare, Chicago, IL, USA) and SepMate tubes (STEMCELL Technologies, Vancouver, BC, Canada) according to the manufacturers’ instructions. T cells were stimulated with Dynabeads Human T-Activator CD3/CD28 (Gibco, Grand Island, NY, USA) in the presence or absence of methotrexate (MTX; Sigma-Aldrich, St. Louis, MO, USA). The concentration was selected to approximate the peak serum MTX levels observed during low-dose MTX therapy. B cells were stimulated with 0.5 µg/mL CD40 ligand (CD40L) and 50 ng/mL IL-21 (R&D Systems, Minneapolis, MN, USA) in the presence or absence of MTX.

### 2.2 Flow cytometry analysis

Dead cells were stained using the Zombie UV fixable viability kit (BioLegend, San Diego, CA, USA). Surface and intracellular staining were performed with antibodies listed in the **Supplementary Material**. For cell cycle analysis, S-phase entry was assessed using the Click-IT EdU Pacific Blue Kit and FxCycle Far Red (Invitrogen, Waltham, MA, USA). Cells were incubated with 2.5 µM EdU for 3 hours, fixed, permeabilized, and stained according to manufacturer protocols. For proliferation assays, PBMCs were labeled with 0.2 µM CellTrace Far Red (Invitrogen) in pre-warmed HBSS, incubated at 37 °C for 10 min, and washed. The division index was calculated as the total number of divisions divided by the initial cell count. For apoptosis assays, Annexin V Alexa Fluor 647 (BioLegend) was used following standard protocols. Flow cytometry data were acquired using BD Symphony A3 and LSRFortessa instruments (BD Biosciences, San Diego, CA, USA) and analyzed with FlowJo v10.10.0 (FlowJo LLC, Ashland, OR, USA, RRID: SCR_008520) (**Supplementary Figure 1**).

### 2.3 Liquid chromatography-mass spectrometry analysis

Intracellular MTX-PG (MTX-PG1, MTX-PG2, and MTX-PG3) levels were quantified by LC-MS/MS as previously described (30). Briefly, T cells and B cells were purified using the Pan T Cell Isolation Kit or Pan B Cell Isolation Kit (Miltenyi Biotec, Bergisch Gladbach, NRW, Germany), counted, and kept frozen until analysis. The frozen cell pellets were resuspended in 50 µL of Millipore water with 10 µL of a 10 µM MTX-PG1–3 isotope-labeled internal standard (Schircks Laboratories, Jona, Switzerland), followed by deproteinization with 100 µL of 16% perchloric acid (Sigma-Aldrich). The mixture was vortexed, centrifuged at 13,000 × g for 10 min, and the supernatant was applied to HLB 96-well plates (Waters, Milford, MA, USA). After sample loading, wells were washed and eluted with 150 µL of 2.5% ammonium hydroxide in methanol (Sigma-Aldrich). The eluates were evaporated under nitrogen gas, reconstituted in 30 µL of 2.5% formic acid in 20% methanol, and 10 µL was injected into an LC-MS/MS system (Agilent Technologies, Santa Clara, CA, USA) for analysis. Multiple reaction monitoring parameters, internal standards, intra- and inter-day variability are summarized in **Supplementary Tables S2** and **S3**.

### 2.4 mRNA expression analysis

Gene expression profiles (GSE197067) were obtained from the Gene Expression Omnibus (GEO) database (http://www.ncbi.nlm.nih.gov/geo/). This dataset includes RNA sequencing of human T cells stimulated with anti-CD3/CD28 beads over time, along with unstimulated controls. RNA-seq read counts were normalized using DESeq2 (v1.46.0).

### 2.5 Western blot

Cellular proteins were extracted from isolated T cells using RIPA lysis buffer (Thermo Scientific, Waltham, MA, USA) supplemented with protease and phosphatase inhibitor cocktail (Thermo Scientific). Protein concentrations were determined using a Bradford assay (Bio-Rad, Hercules, CA, USA), and samples were denatured in SDS-PAGE loading buffer (Biosesang, Gyeonggi-do, Korea). Proteins were separated on 4-12% Bis-Tris gels (Invitrogen) and transferred onto polyvinylidene difluoride (PVDF) membranes (Invitrogen). The membranes were blocked with 5% bovine serum albumin (BSA) buffer for 1 hour at room temperature, followed by incubated with anti-RFC1 (1:1000, #ab229229, Abcam, Cambridge, UK), anti-FPGS (1:2000, #ab184564, Abcam), anti-GGH (1:500, #ab138495, Abcam), and anti-β-Actin (1:5000, #3700, Cell Signaling Technology, RRID: AB_2242334) primary antibodies overnight at 4 °C. After washing, membranes were incubated with anti-rabbit (1:2000, #ab6721, Abcam, RRID: AB_955447) or anti-mouse (1:100000, #G-21040, Invitrogen, RRID: AB_2536527) HRP conjugated secondary antibodies for 1 hour at room temperature. Target signal was detected by Enhanced Peroxidase Detection kit (Elpis Biotech, Daejeon, Korea) using Fusion FX (Vilber Lourmat, ZAC de Lamirault, France).

### 2.6 Enzyme-linked immunosorbent assay

B cells were stimulated with CD40L and IL-21 with MTX treatment at defined time points. Cells were washed with HBSS at 96 hours, and supernatants were collected at 144 hours, centrifuged, and stored at -80°C. Total human IgG levels were measured using Human IgG Total ELISA Kit (Invitrogen) according to manufacturer’s instructions. Briefly, standards and 1:50 diluted samples were added to antibody-coated wells along with a diluted HRP-conjugated antibody. Following incubation and washing, TMB substrate solution was added and incubated at room temperature for 20 min. The reaction was stopped, and optical density was measured at 450 nm using Infinite M Nano microplate reader (Tecan, Männedorf, Zurich, Switzerland). Concentrations were determined using a 5-parameter curve fit standard curve.

### 2.7 Time-specific effects of MTX on vaccine response

A *post hoc* analysis of the two randomized controlled trials that investigated the effect of MTX on responses to seasonal influenza vaccination (4, 6). Briefly, in the first trial, patients with RA receiving a stable dose of MTX were randomized to hold 2 weekly doses of MTX or to continue MTX after quadrivalent influenza vaccine containing H1N1, H3N2, B-Yamagata, and B-Victoria (www.clinicaltrials.gov; trial registration number NCTNCT02897011). In the second clinical trial, RA patients were randomized to hold one weekly dose vs 2 weekly doses of MTX after quadrivalent influenza vaccine (www.clinicaltrials.gov; trial registration number NCT05069714).

Patients were stratified according to the interval (days) between vaccination and MTX resumption. Vaccine responses were evaluated based on the number of influenza antigens eliciting a positive antibody response, according to MTX timing after vaccination. A positive response was defined as a ≥4-fold increase in antibody titer at 4 weeks post-vaccination relative to baseline.

### 2.8 Statistical analysis

Data are presented as mean ± standard deviation (SD). Group comparisons were performed using a two-way ANOVA or a mixed-effects models appropriate, with time and treatment as factors. Mixed-effects models were used when data were missing. *Post hoc* analyses were conducted using the Bonferroni correction for multiple comparisons. A p-value of less than 0.05 was considered statistically significant. All statistical analyses were performed using GraphPad Prism 8 software (GraphPad Software, San Diego, CA, USA, RRID: SCR_002798).

## 3 Results

### 3.1 MTX suppressed T cell activation and proliferation and induced late S-phase arrest and apoptosis

**Figure 1:**
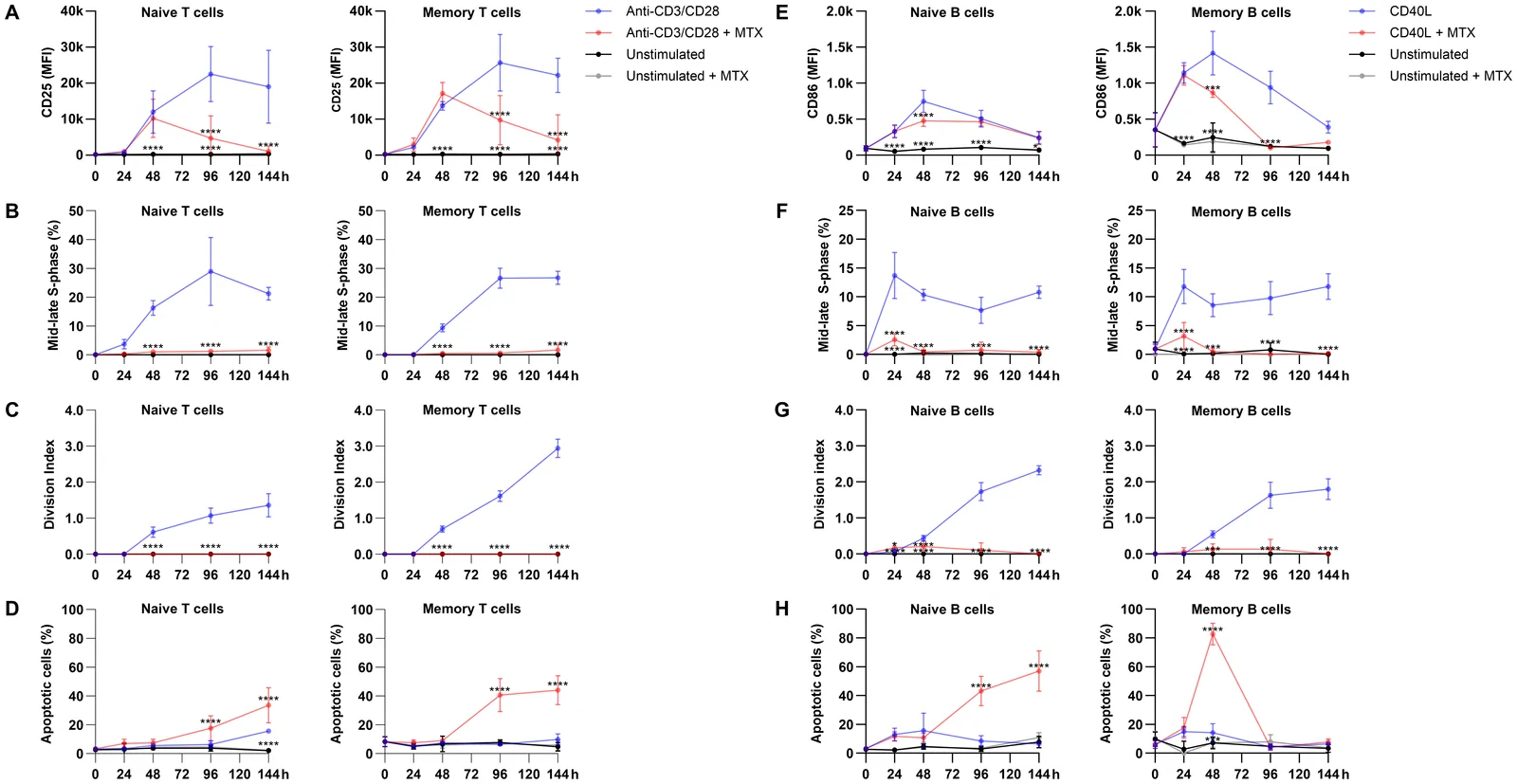
MTX suppressed lymphocyte activation, proliferation, and survival. T cells and B cells from healthy donors (n = 4) were stimulated with anti-CD3/CD28 beads and CD40L/IL-21, respectively, in the presence or absence of 2 μM MTX. **(A, E)** Mean fluorescence intensity (MFI) of CD25 on T cells and CD86 on B cells. **(B, F)** Percentage of cells in mid–late S phase, assessed by EdU/FxCycle cell cycle assay. **(C, G)** Division index measured by CellTrace dilution. **(D, H)** Percentage of apoptotic cells determined by Annexin V staining. Data are presented as mean ± SD. Statistical significance: **P < 0.01, ***P < 0.001, ****P < 0.0001. Multiple comparisons were performed using Bonferroni correction.

CD4^+^ T cells were stimulated with anti-CD3/CD28 beads in the presence or absence of MTX. Naïve T cells (TN) upregulated the early activation marker CD25 by 48 hours post-stimulation, with expression peaking at 96 hours and remaining elevated through 144 hours (**Figure 1A**; **Supplementary Figure 1**). MTX did not affect CD25 expression at 48 hours but led to a progressive decline thereafter. The memory T (TM) cells showed similar patterns of CD25 expression. The late activation marker CD38 followed a comparable trajectory, peaking at 96 hours, and was significantly suppressed by MTX (**Supplementary Figure 2**).

At 48 hours post-stimulation, 67.8 ± 13.2% of TN cells entered the S-phase with 16.3 ± 2.5% progressing to the mid-late S-phase (**Figure 1B**). Accordingly, TN cells demonstrated increasing proliferation over time (**Figure 1C**). Only 15.7 ± 1.7% of TN cells underwent apoptosis at 144 hours (**Figure 1D**). In contrast, MTX-treated cells showed significantly reduced proliferation compared to the MTX-untreated cells: 54.0 ± 9.5% entered S phase (vs. 67.8 ± 13.2%, *P* = 0.014), and only 1.0 ± 0.3% reached mid-late S phase (vs. 16.3 ± 2.5%, *P* < 0.0001), resulting in a division index of 0. MTX treatment increased apoptosis at 96 hours (17.6 ± 8.6% vs. 6.3 ± 2.6%, *P* < 0.0001) compared to MTX-untreated. TM cells exhibited higher proliferation rate but were more susceptible to MTX-induced apoptosis compared to TN cells. Non-stimulated T cells neither proliferate nor undergo apoptosis regardless of MTX treatment, highlighting the activation-dependent effects of MTX.

B cells were stimulated with CD40L and IL-21 in the presence or absence of MTX. In the absence of MTX, activated B cells upregulated the activation marker CD86, entered the cell cycle by 24 hours, and exhibited increased proliferation by 48 hours, consistent with the short G1 entry time of B cells compared to T cells (**Figures 1E–H**). MTX significantly inhibited CD86 upregulation in naïve B (BN) cells at 48 hours (476.5 ± 77.9 vs. 746.0 ± 155.1 MFI, *P* < 0.0001). Entry into mid-to-late S phase at 24 hours was also markedly reduced by MTX (2.6 ± 1.1% vs. 13.7 ± 4.0%, *P* < 0.0001), followed by diminished proliferation at 96 hours (division index, 0.1 ± 0.2 vs. 1.7 ± 0.3, *P* < 0.0001) and increased apoptosis (43.1 ± 10.2% vs. 8.3 ± 3.7%, *P* < 0.0001). These inhibitory effects were even more pronounced in memory B cells. Non-stimulated B cells were unaffected by MTX.

### 3.2 Activation phase-dependent sensitivity to MTX exposure

**Figure 2:**
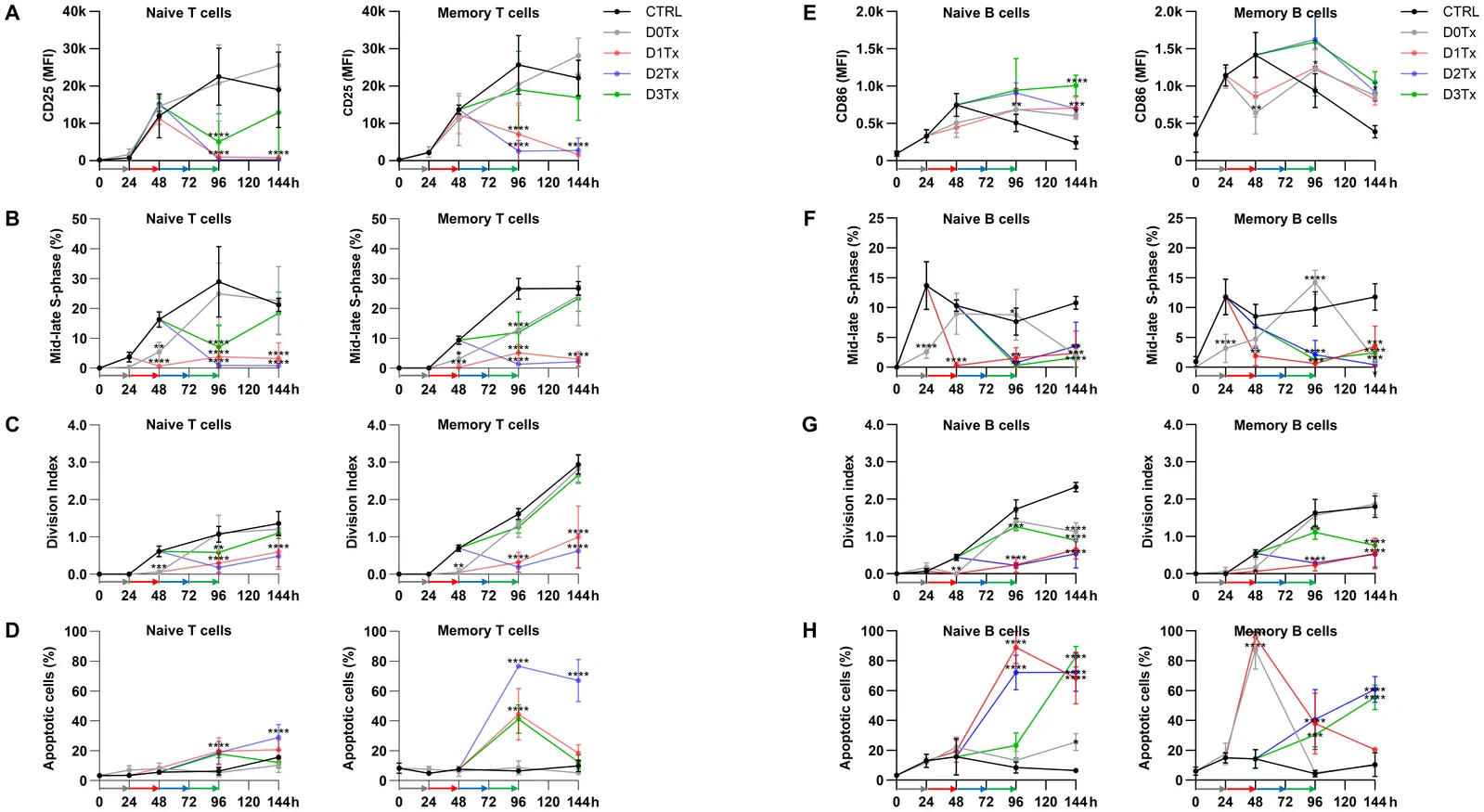
MTX exerted activation-phase-specific effects on lymphocytes with rapid on and off effect. T cells and B cells from healthy donors (n = 4) were stimulated with anti-CD3/CD28 beads and CD40L/IL-21, respectively, and treated with a 24-hour pulse of methotrexate (MTX, 2 μM) at defined activation time points: Day 1 (D1Tx; 0–24 hours), Day 2 (D2Tx; 24–48 hours), Day 3 (D3Tx; 48–72 hours), and Day 4 (D4Tx; 72–96 hours). **(A, E)** Mean fluorescence intensity (MFI) of CD25 on T cells and CD86 on B cells. **(B, F)** Percentage of cells in mid–late S phase, assessed by EdU/FxCycle cell cycle assay. **(C, G)** Division index measured by CellTrace dilution. **(D, H)** Percentage of apoptotic cells determined by Annexin V staining. Data are shown as mean ± SD. Statistical significance: *P < 0.05, **P < 0.01, ***P < 0.001, ****P < 0.0001. Multiple comparisons were corrected using the Bonferroni method.

To assess whether MTX sensitivity varies by activation phase, CD4^+^ T cells and B cells were treated with a 24-hour MTX pulse on Day 1 (D1Tx: 0–24 h), Day 2 (D2Tx: 24–48 h), Day 3 (D3Tx: 48–72 h), or Day 4 (D4Tx: 72–96 h) after stimulation. In TN, early MTX treatment (D1Tx) had minimal effects on activation or proliferation (**Figures 2A–C**). However, treatment on day 2 (D2Tx) or day 3 (D3Tx) significantly suppressed both activation markers (328.3 ± 30.2 MFI vs. 22,488.3 ± 7,656.3 MFI, *P* < 0.0001) and proliferation (0.2 ± 0.1 vs. 1.1 ± 0.2, *P* < 0.0001) compared to MTX-untreated at 96 hours, accompanied by increased apoptosis (18.7 ± 7.5% vs. 6.3 ± 2.6%, *P* < 0.0001) (**Figures 2A, C, D**). D4Tx resulted in transient suppression of activation (5,070.5 ± 7,519.2 MFI vs. 22,488.3 ± 7,656.3 MFI, *P* < 0.0001) and a moderate increase in apoptosis (17.9 ± 2.6% vs. 6.3 ± 2.6%, *P* < 0.0001) at 96 hours, both of which were partially reversed by 144 hours. TM cells exhibited similar temporal patterns but were more sensitive to MTX than TN cells.

B cells displayed a distinct temporal response to MTX. A 24-hour MTX treatment on Day 1 (D1Tx) significantly inhibited entry into mid-late S phase (2.6 ± 1.1% vs. 13.7 ± 4.0%, *P* < 0.0001) at 24 hours and proliferation (0.0 ± 0.0 vs. 0.4 ± 0.0, *P* = 0.0009) by 48 hours, compared to MTX-untreated BN cells (**Figures 2F, G**). However, both cell-cycle entry and proliferation partially recovered following MTX washout. In contrast, MTX exposure on Day 2, Day 3, or Day 4 resulted in sustained suppression of mid-late S-phase entry and proliferation, along with increased apoptosis within 24 hours of treatment (**Figures 2E–H**). These inhibitory effects partially recover after MTX washout. Consistent with this, a 24-hour MTX exposure on Day 1 (D1Tx) did not reduce the number of antibody-secreting cells (ASCs), whereas exposure on Days 2–4 significantly decreased ASCs (**Supplementary Figure 3**).

### 3.3 Intracellular MTX accumulation is activation-dependent

**Figure 3:**
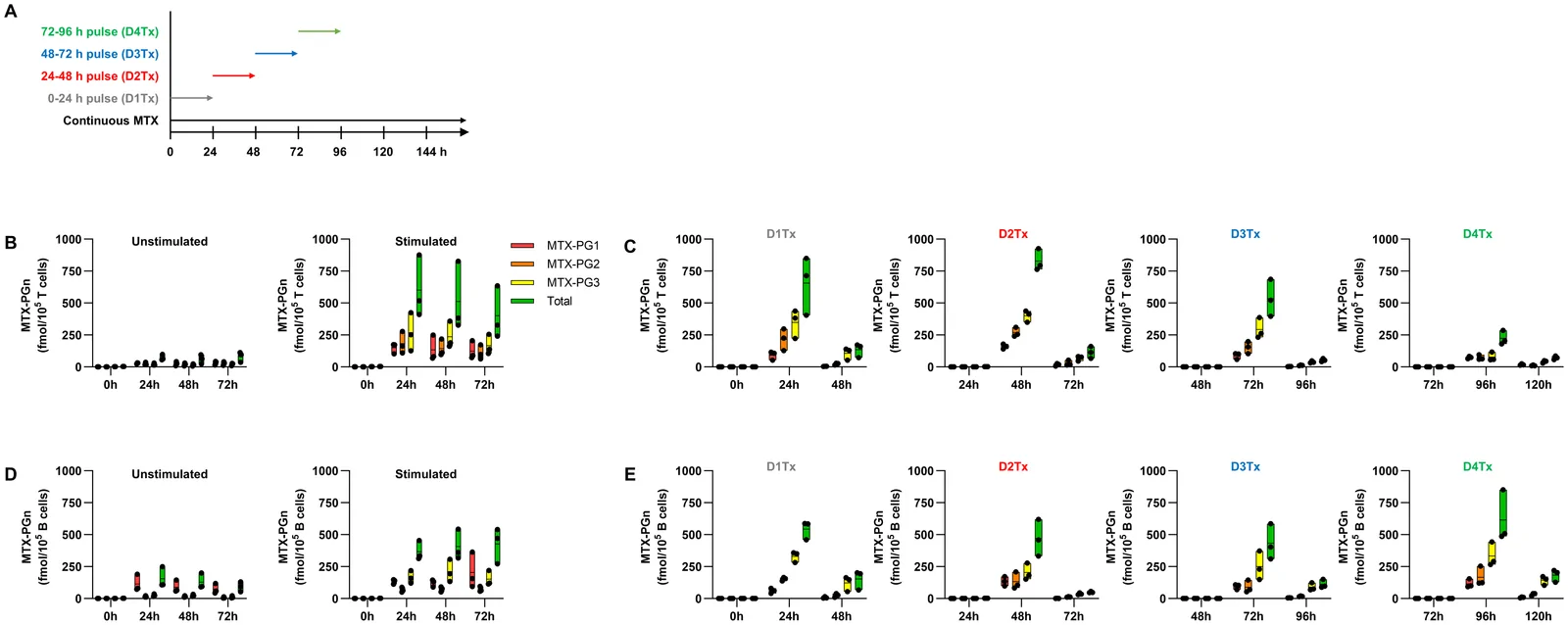
Intracellular MTX levels during lymphocyte activation. **(A)** Schematic overview of the experimental design. T cells and B cells from healthy donors (n = 3) were stimulated and treated with 2.0 μM methotrexate (MTX). Intracellular levels of methotrexate polyglutamates (MTX-PGs) were quantified using liquid chromatography–mass spectrometry. **(B, D)** Intracellular MTX-PG levels in unstimulated and stimulated T cells and B cells under continuous MTX exposure. **(C, E)** Intracellular MTX-PG levels in stimulated T cells and B cells following a 24-hour pulse MTX (2.0 μM) treatment at the indicated time points. Data are presented as individual data points, with the box representing the range and the central line indicating the mean.

T cells were stimulated with anti-CD3/CD28 beads and treated with MTX at defined time points (**Figure 3A**). In unstimulated, resting T cells, intracellular MTX polyglutamate (MTX-PG) levels were low at 80.8 ± 40.2 fmol/10^5^ cells (**Figure 3B**). Continuous MTX exposure during activation resulted in a significant rise in intracellular MTX-PG levels, reaching 601.6 ± 243.4 fmol/10^5^ cells by 24 hours (*P* = 0.002), with MTX-PG3 as the predominant metabolite (**Figure 3B**). Following a 24-hour MTX pulse on Day 1 (D1Tx), intracellular MTX-PG levels peaked at 655.9 ± 228.4 fmol/10^5^ cells at 24 hours and declined to 135.0 ± 54.0 fmol/10^5^ cells within 48 hours after treatment (**Figure 3C**). Similarly, Day 2 treatment (D2Tx) resulted in a peak concentration of 828.2 ± 86.3 fmol/10^5^ cells at 24 hours, decreasing to 113.8 ± 46.5 fmol/10^5^ cells by 48 hours. Day 3 treatment (D3Tx) yielded a peak of 534.7 ± 145.6 fmol/10^5^ cells at 24 hours, declining to 53.0 ± 11.5 fmol/10^5^ cells at 48 hours. In contrast, Day 4 treatment (D4Tx) resulted in substantially lower MTX-PG accumulation, reaching 223.4 ± 55.3 fmol/10^5^ cells at 24 hours and decreasing to 70.9 ± 13.9 fmol/10^5^ cells by 48 hours.

In resting B cells, intracellular MTX-PG levels reached 154.5 ± 81.5 fmol/10^5^ cells at 24 hours (**Figure 3D**). Upon activation, MTX-PG levels increased rapidly to 367.1 ± 76.4 fmol/10^5^ cells at 24 hours and remained stable thereafter. A 24-hour MTX pulse on Day 1 (D1Tx) resulted in peak MTX-PG levels of 543.4 ± 72.9 fmol/10^5^ cells at 24 hours, followed by a decline to 153.8 ± 75.7 fmol/10^5^ cells at 48 hours post-treatment. Pulse treatment on Days 2–4 showed similar kinetics (**Figure 3E**).

### 3.4 Activation induces dynamic changes in MTX metabolic enzymes and transporters

**Figure 4:**
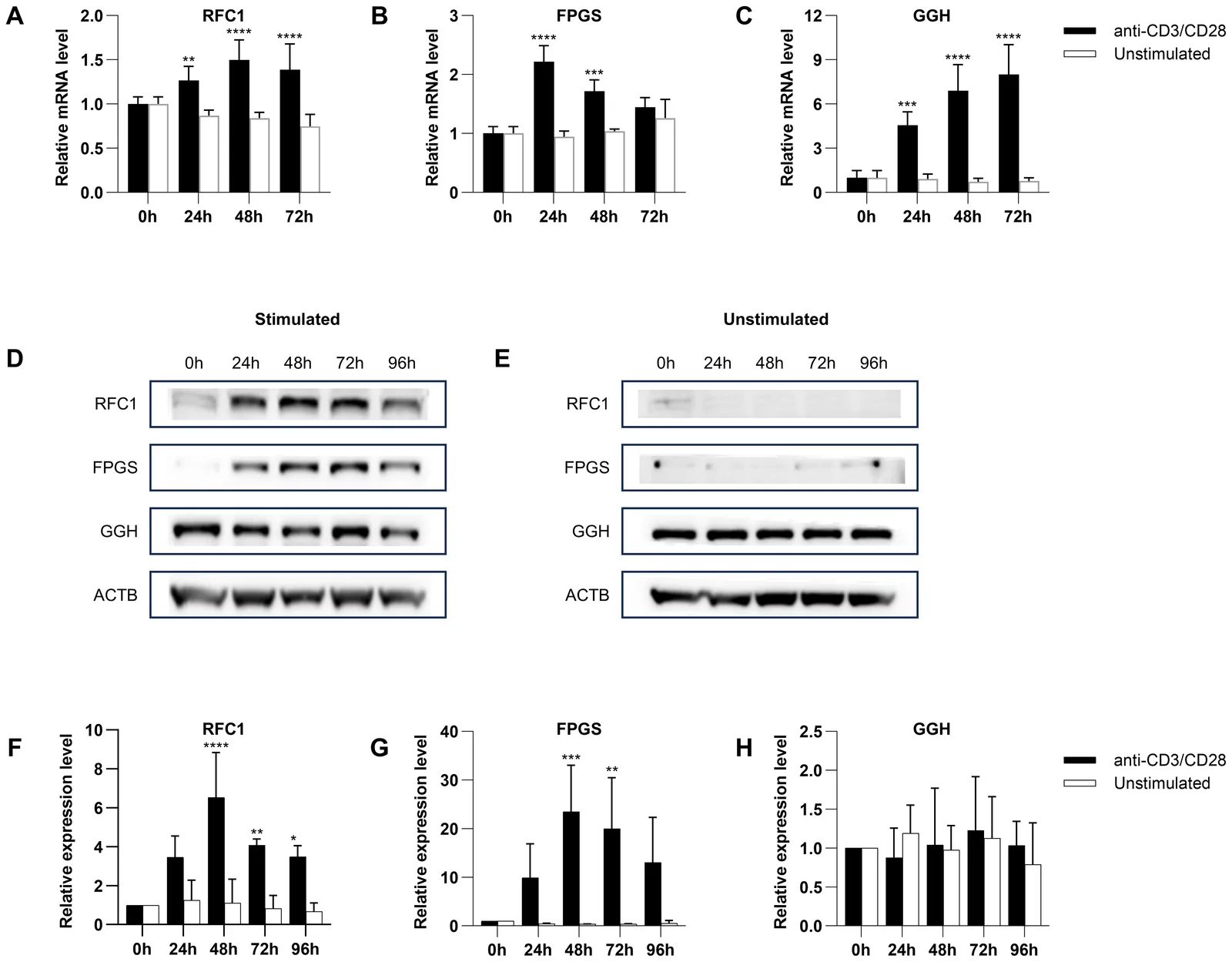
Expression dynamics of MTX transport and metabolism–related genes during T cell activation. **(A–C)** Relative mRNA expression level of RFC1 (reduced folate carrier 1), FPGS (folylpolyglutamate synthase), and GGH (γ-glutamyl hydrolase) in T cells (n = 4) stimulated with anti-CD3/CD28 beads or left unstimulated. **(D–H)** Protein expression analysis in T cells from healthy donors (n = 3). **(D–E)** Representative immunoblots of RFC1, FPGS, and GGH in stimulated and unstimulated conditions. **(F–H)** Quantification of relative protein expression levels. Data are presented as mean ± SD. Statistical significance: *P < 0.05, **P < 0.01, ***P < 0.001, ****P < 0.0001. Multiple comparisons were corrected using the Bonferroni method.

To elucidate the mechanism underlying activation-dependent MTX metabolism, mRNA and protein expression of key folate pathway transporters and enzymes were assessed in CD4^+^ T cells following activation. mRNA expression of the folate transporter RFC1 increased rapidly within 24 hours post-activation and remained stable thereafter (**Figure 4A**). In contrast, FPGS expression peaked at 24 hours and subsequently declined (**Figure 4B**), whereas GGH expression gradually increased, reaching an ~8.0-fold increase by 72 hours (**Figure 4C**).

At the protein level, RFC1 expression peaked at 48 hours post-activation, increasing 6.5-fold relative to unstimulated cells (*P* < 0.0001), and then gradually declined (**Figures 4D–H**). FPGS protein levels mirrored the mRNA expression pattern. In contrast, GGH protein expression remained largely unchanged. Expression of ATP-binding cassette (ABC) transporters, as well as extracellular purine-regulating enzymes CD39 and CD73, did not change significantly following activation (**Supplementary Figure 4**).

### 3.5 Vaccination response according to the days between vaccination and MTX re-uptake

**Figure 5:**
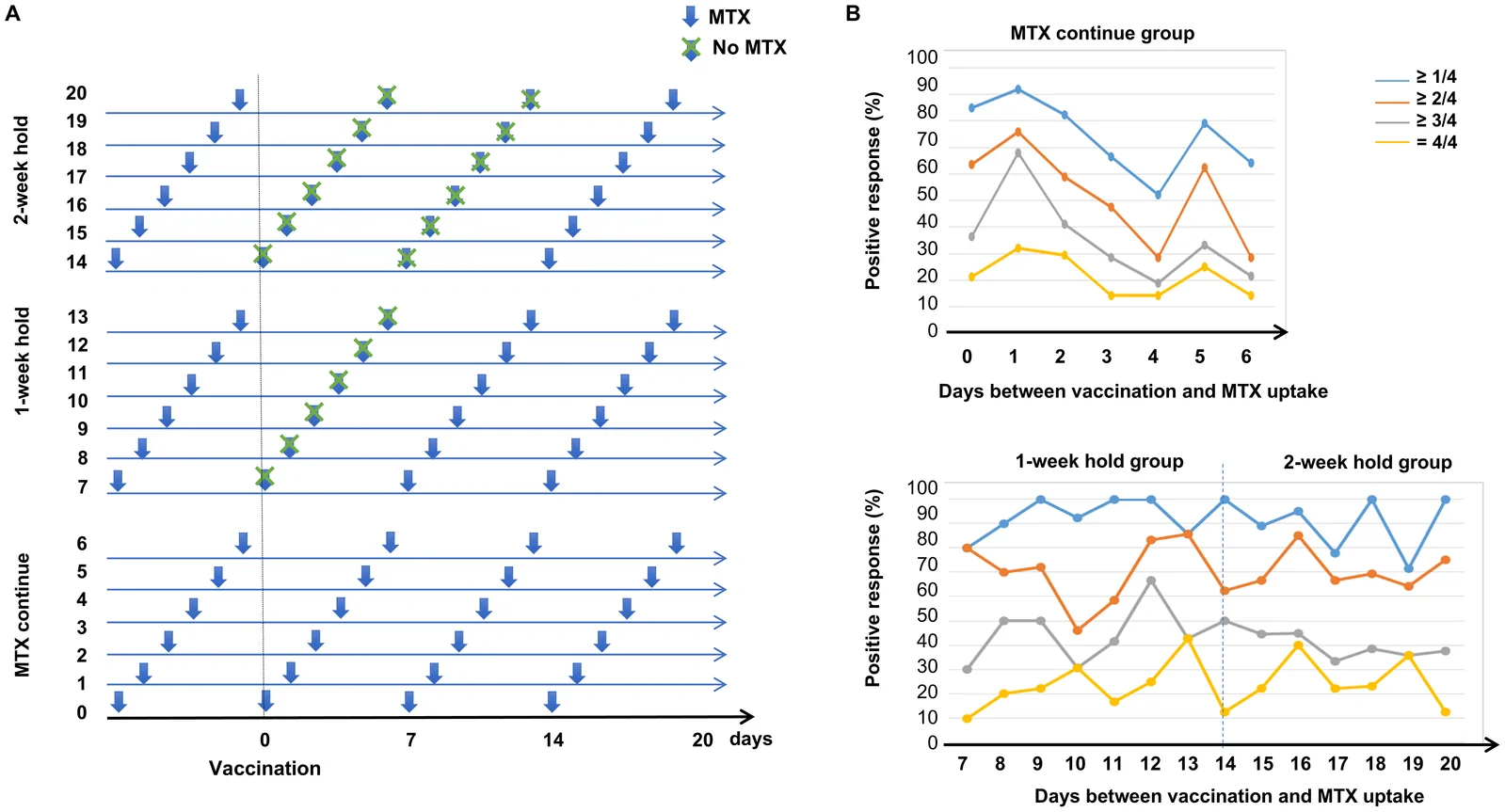
Vaccine response according to the time between the vaccination and MTX re-uptake. **(A)** A total of 318 patients with rheumatoid arthritis (RA) were stratified by the interval (days) between vaccination and MTX re-uptake. The 0-day group indicates patients who resumed MTX on the day of vaccination. **(B)** Proportion of patients achieving a positive antibody response to ≥1, ≥2, ≥3, or all 4 antigens included in the quadrivalent seasonal influenza vaccine, according to the interval between vaccination and MTX re-uptake.

The baseline characteristics and MTX doses were comparable across subgroups stratified by the timing of MTX resumption after vaccination (**Table 1**). Notably, MTX administration on the day of vaccination (Day 0) or Day 1 thereafter did not significantly impair vaccine immunogenicity. In contrast, MTX administration on Day 2 post-vaccination was associated with reduced immunogenicity, with the lowest responses observed when MTX was resumed on Days 3–4 (**Figure 5B**, upper panel). Delaying MTX resumption until 7 or more days post-vaccination induced robust vaccine responses (**Figure 5B**, lower panel). The geometric mean fold rises in antibody titers are summarized in **Supplementary Table 4**.

**Table 1:** Baseline characteristics by days between vaccination and MTX re-uptake.

Patient characteristics	Days 0-6 (n = 155)	Days 7-13 (n = 82)	Days 14-20 (n = 81)
Female (%)	128 (82.6)	74 (90.2)	69 (85.2)
Age, years	52.2 ± 9.5	57.1 ± 12.3	58.8 ± 10.9
Duration of RA, years	6.9 ± 6.5	11.1 ± 7.9	9.8 ± 6.8
Body mass index, kg/m^2^	23.3 ± 3.3	23.1 ± 3.8	23.1 ± 3.7
Diabetes mellitus	8 (5.2)	4 (4.9)	7 (8.6)
Hypertension	24 (15.5)	29 (35.4)	20 (24.7)
Smoking, ever smoked	28 (18.1)	13 (15.9)	11 (13.6)
RF positivity	120 (77.4)	64 (78)	58 (71.6)
Anti-CCP positivity	104 (86)	67 (82.7)	57 (74)
DAS28-CRP	2.2 ± 0.9	2.2 ± 0	2.2 ± 0
Treatment			
GC	81 (52.3)	34 (41.5)	37 (45.7)
Mean GC dose, mg/day	1.8 ± 2.1	3.8 ± 1.9	4.4 ± 1.9
MTX	155 (100)	82 (100)	81 (100)
MTX dose, mg/week	13.3 ± 3.4	12.6 ± 0	12.8 ± 0
Sulfasalazine	8 (5.2)	4 (4.9)	7 (8.6)
Hydroxychloroquine	35 (22.6)	20 (24.4)	22 (27.2)
Leflunomide	33 (21.3)	17 (20.7)	17 (21)
Tacrolimus	2 (1.3)	1 (1.2)	2 (2.5)
Biological DMARDs			
TNF inhibitor	11 (7.1)	10 (12.2)	5 (6.2)
Abatacept	4 (2.6)	3 (3.7)	1 (1.2)
Tocilizumab	0 (0)	1 (1.2)	3 (3.7)
Rituximab	1 (0.6)	0 (0)	0 (0)
JAK inhibitor	0 (0)	2 (2.4)	6 (7.4)

The data are mean (SD) or number (%).

Anti-CCP, anti-cyclic citrullinated peptide; CRP, C-reactive protein; DAS28, Disease Activity Score in 28 joints; DMARDs, disease-modifying antirheumatic drugs; GC, glucocorticoids; JAK, Janus kinase; MTX, methotrexate; RA, rheumatoid arthritis; RF, rheumatoid factor.

## 4 Discussion

In this study, MTX suppressed immune responses in T and B cells in a cell activation-dependent manner. MTX induced cell cycle arrest in the mid-to-late S-phase and promoted apoptosis in activated but not resting lymphocytes. A 24-hour MTX pulse exerted immunosuppressive effects lasting 1–2 days, paralleling the rapid fluctuations in intracellular MTX levels regulated by dynamic expression of MTX transporters and metabolic enzymes during activation. In patients with RA, MTX administration on Days 3–4 following vaccination markedly impaired immunogenicity, whereas delaying MTX for 1 week had minimal suppressive effects. Together, these findings suggest that the short-lived immunosuppressive effects of MTX on activated lymphocytes underlie the rapid restoration of vaccine responses after brief MTX interruption in patients on chronic MTX therapy.

MTX has been considered to have long biological half-life based on clinical observations; joint symptoms typically improve within 3–6 weeks after treatment initiation, whereas disease flares occur more than 2 weeks after discontinuation (8, 9). Although MTX has a short plasma half-life of approximately 3–4 hours and is largely cleared from the circulation within 24 hours, it is retained intracellularly as polyglutamated metabolites (MTX-PGs), which accounts for its long half-life (12, 16, 17). Indeed, intracellular MTX-PG levels in erythrocytes accumulate over time and correlate with therapeutic efficacy in RA patients (20–22). Accordingly, MTX is administrated once weekly as a “mini pulse” of 15–25 mg/week (23, 24). However, our previous randomized clinical trials showed that temporarily withholding MTX for only 1–2 weeks after vaccination effectively restored vaccine response to seasonal influenza vaccines in RA patients, while MTX discontinuation before the vaccination did not (4, 6, 25). This paradox prompted investigation into the time-dependent immunosuppressive effects of MTX on vaccine response.

MTX suppressed early T cell activation. Although MTX did not prevent entry into the cell cycle, it induced arrest in the mid–late S phase followed by apoptosis, consistent with its inhibition of *de novo* purine and pyrimidine synthesis required for DNA replication during S-phase (12, 19). Memory T cells, which have higher proliferative capacity, were more susceptible to MTX-induced apoptosis (26, 27). To model the once-weekly dosing regimen used in RA, activated T cells were exposed to a 24-hour MTX pulse at defined time points. During low-dose MTX therapy, serum MTX concentrations peak at below 2 μM within 2 hours of administration, decline rapidly to approximately 0.1 μM by 6 hours, and become negligible within 24–48 hours. Accordingly, 2 μM MTX was selected as a conservative *in vitro* exposure condition approximating peak serum concentrations.

MTX exerted maximal immunosuppressive effects when administered during the early proliferative phase (24–72 hours post-activation), whereas treatment during the first 24 hours had minimal impact and exposure after 72 hours was substantially less effective. These findings indicate that MTX exerts activation phase-dependent effects, likely reflecting dynamic changes in intracellular drug levels. Indeed, intracellular MTX-PG levels increased rapidly within 24 hours of exposure but declined sharply by 48 hours. Strikingly, MTX uptake was markedly lower when administered at later stage (72–96 hours) of activation. Resting T cells showed minimal intracellular MTX accumulation. Therefore, intracellular MTX pharmacokinetics are closely tied to T cell activation state. A similar kinetic pattern was observed in B cells, with rapid accumulation followed by washout after 24 hours of MTX exposure. However, in contrast to activated T cells, activated B cells retained a greater capacity to accumulate MTX at later time points (e.g., Day 4 of activation). This likely accounts for the relatively preserved sensitivity of B cells to MTX treatment at this stage, particularly with respect to proliferation and apoptosis.

To investigate the activation-dependent kinetics of MTX, we examined the expression of key MTX transporters and metabolic enzymes in activated T cells. RFC1, the primary MTX influx transporter, was upregulated within 24 hours of activation and remained elevated thereafter. FPGS, which mediates polyglutamation, was transiently increased during early activation but declined at later time points, whereas GGH, responsible for MTX deglutamation, remained largely unchanged. This dynamic expression pattern supports enhanced MTX uptake and retention during early activation, followed by reduced accumulation at later stages. The low baseline RFC1 expression in resting T cells explains limited MTX uptake and minimal functional response. Taken together, these findings indicate that intracellular MTX kinetics in T cells are rapid and activation dependent, characterized by swift uptake and clearance, in contrast to the prolonged retention observed in erythrocytes (20–22). Analyzed of the publicly available dataset further showed that the expression ABC transporters as well as CD39 and CD73, the enzymes responsible for extracellular conversion of ATP to anti-inflammatory adenosine remained largely unchanged following T cell activation (12). Although these findings do not support major activation-dependent transcriptional regulation of these pathways, they do not exclude potential contributions of changes in protein expression or functional activity to MTX pharmacokinetics.

We next examined the impact of MTX timing on vaccine responses in patients with RA receiving seasonal influenza vaccination. Consistent with the *in vitro* findings, MTX administration on the day of vaccination or 1 day thereafter had minimal effects on immunogenicity. In contrast, MTX administration on Days 3–4 significantly suppressed vaccine responses. This suppressive effect diminished when MTX was administered on Days 5–6 and was negligible when given ≥1 week after vaccination, supporting clinical evidence that withholding MTX for at least 1 week restores vaccine immunogenicity (4, 28, 29). Consistent with these observations, a 24-hour MTX pulse administered on Days 2–4 of B cell activation *in vitro* markedly reduced the number of antibody-secreting cells at Day 6, highlighting a critical window during which MTX most effectively impairs humoral responses. Taken together, these findings suggest that the short intracellular half-life of MTX contributes to the rapid recovery of vaccine responses following brief MTX interruption in patients receiving chronic therapy. However, whether this rapid kinetics extends to tissue-resident cells, such as those in inflamed joints, remains to be determined.

One limitation of this study is the absence of direct longitudinal measurement of MTX levels in patient immune cells following vaccination. Although erythrocyte MTX-PG levels are commonly used to monitor MTX exposure, they may not reflect dynamic intracellular concentrations in activated T and B cells. Indeed, MTX levels in leukocytes can be up to 10-fold higher than in erythrocytes, and activated cells may accumulate even greater amounts of MTX (30). Therefore, direct measurement of MTX in immune cells would provide a more accurate assessment of its pharmacologic effects and toxicity. In addition, the impact of genetic polymorphisms affecting MTX uptake and metabolism, including polyglutamation, on inter-individual and ethnic variability in therapeutic response and toxicity warrants further investigation (22). Furthermore, we did not assess adenosine production, a key mediator with potent anti-inflammatory effects across immune cell types (31). Adenosine is generated, in part, through enzymatic degradation of extracellular ATP by CD39 and CD73, whose expression can be modulated by B cell–activating factor (BAFF). MTX has been shown to attenuate vaccine responses and reduce anti-drug antibody formation in RA patients with elevated serum BAFF levels, suggesting a functional interaction between MTX and BAFF signaling pathways (32, 33). These effects are independently of MTX’s folate-antagonist activity. Consistent with this, the early and late activation markers CD25 and CD38 were suppressed and swiftly recovered after MTX-pulse treatment, further supporting a distinct immunoregulatory mechanism linked to MTX-BAFF-adenosine axis. Furthermore, the *in vitro* experiments were performed using lymphocytes from a limited number of healthy donors rather than patients with RA receiving chronic MTX. Disease-related immune alterations and prior MTX exposure may influence lymphocyte activation and MTX pharmacodynamics, limiting a direct extrapolation of our findings to patients with RA. Further studies evaluating the effects of MTX on the lymphocytes from treatment-naive RA patients would be of particular interest. Finally, the analysis of vaccine responses according to the interval between vaccination and MTX re-uptake should be considered exploratory and descriptive, given the limited sample size within each subgroup.

In conclusion, MTX exerts activation-phase-specific effects on T cells, driven by dynamic intracellular pharmacokinetics that are closely linked to the activation status of the cell. These findings suggest that the relatively short intracellular half-life may contribute to the rapid restoration of vaccine responses following brief MTX discontinuation, although this mechanism remains to be determined *in vivo*. Further studies are warranted to determine whether optimizing the timing of MTX administration may enhance immune responses during immunologic challenges, such as infection or surgery, in RA patients on chronic MTX therapy.

## Data Availability

Gene expression profiles (GSE197067) were obtained from the Gene Expression Omnibus (GEO) database (http://www.ncbi.nlm.nih.gov/geo/). This dataset includes RNA sequencing of human T cells stimulated with anti-CD3/CD28 beads over time, along with unstimulated controls. RNA-seq read counts were normalized using DESeq2 (v1.46.0). The data supporting the findings of this study are available from the corresponding author upon reasonable request.
