# Supplementary material for: Rapid on–off effects of methotrexate on lymphocytes may facilitate recovery of suppressed vaccine immunogenicity after its short-term methotrexate discontinuation in rheumatoid arthritis
Source: Front Immunol. 2026 Aug 7;17:1898581. doi: 10.3389/fimmu.2026.1898581 (PMC13493376; doi:10.3389/fimmu.2026.1898581)

Supplementary Material

Rapid on–off effects of methotrexate on lymphocytes may facilitate recovery of suppressed vaccine immunogenicity after its short-term MTX methotrexate discontinuation in rheumatoid arthritis.

Supplementary Methods

Reagents

BV711 anti-human CD3 antibody (344838, RRID:AB_2565827), Spark UV 387 anti-human CD4 antibody (344686, RRID:AB_2922562), BV510 CD8 anti-human CD8 antibody (344732, RRID:AB_2564624), PE anti-human CCR7 antibody (353204, RRID:AB_10913813), BV421 anti-human CD25 antibody (302630, RRID:AB_11126749), BV785 anti-human CD38 antibody (303530, RRID:AB_2565893), Annexin V AF647 (640943) and Zombie UV (423107) were obtained from BioLegend (San Diego, CA, USA). BUV737 anti-human CD8 antibody (612754, RRID:AB_2870085) and FITC anti-human CD45RA antibody (555488, RRID:AB_395879) were obtained from BD Bioscience (San Diego, CA, USA). CellTrace Far Red (C34572), EdU Pacific Blue (C10636) and FxCycle Far Red (F10348) were obtained from Invitrogen (Waltham, MA, USA).

Supplementary Table S1. Baseline characteristics of donors and corresponding experiments.

Donor ID	Age	Sex	Experiment
HC1	25	Female	Fig 1, 2, S2 (T cells)
HC2	27	Female	Fig 1, 2, S2 (T cells)
HC3	26	Female	Fig 1, 2, S2 (T cells), Fig 3-B
HC4	28	Female	Fig 1, 2, S2 (T cells)
HC5	22	Female	Fig 1, 2 S3 (B cells)
HC6	30	Female	Fig 1, 2 S3 (B cells)
HC7	26	Female	Fig 1, 2 S3 (B cells), Fig 4
HC8	30	Female	Fig 1, 2 S3 (B cells), Fig 4
HC9	27	Male	Fig 3-B
HC10	26	Male	Fig 3-B, D
HC11	30	Male	Fig 3-C
HC12	26	Male	Fig 3-C, D
HC13	30	Male	Fig 3-C, E
HC14	48	Male	Fig 3-D
HC15	25	Male	Fig 3-E
HC16	30	Male	Fig 3-E
HC17	25	Female	Fig 4

HC = healthy control.

Supplementary Table S2. Multiple reaction monitoring (MRM) parameters for analytes and internal standards.

		Retention time (min)	MRM transition		Collision energy (V)
			Q1	Q3	
Target analyte	MTXPG1	2.0	455.2	308.2	20
	MTXPG2	1.5	584.2	308.2	24
	MTXPG3	1.4	713.3	308.2	40
Internal standard	MTXPG1-d3	2.0	458.3	175.1	36
	MTXPG2-d3	1.5	587.3	311.2	34
	MTXPG3-d3	1.4	716.4	311.3	41

Supplementary Table S3. Intra- and inter-day accuracy and precision for MTX-PG quantification.

The accuracy and precision of MTX-PG1, MTX-PG2, and MTX-PG3 quantification were assessed at three nominal concentrations. Accuracy is expressed as the mean percentage of the nominal concentration, whereas precision is expressed as the coefficient of variation (CV, %) for intra- and inter-day analyses.

Analyte	Nominal concentration (nM)	Intra-day (n=6)		Inter-day (n=11)	
		Accuracy (%)	CV (%)	Accuracy (%)	% CV
MTX-PG1	7.5	98.0	4.9	99.0	4.8
	37.5	95.3	4.0	99.2	6.9
	175	95.7	3.6	97.6	7.5
MTX-PG2	7.5	97.6	4.3	100.5	4.1
	37.5	95.6	3.4	102.0	5.0
	175	97.9	3.0	104.6	3.4
MTX-PG3	7.5	98.3	4.2	98.4	6.0
	37.5	95.6	3.0	98.4	8.2
	175	97.8	2.8	100.7	6.5

Supplementary Table S4. Geometric mean fold rise in antibody titers according to the interval between vaccination and MTX re-uptake.

Days	N	H1N1 GMFR (95% CI)	H3N2 GMFR (95% CI)	Yamagata GMFR (95% CI)	Victoria GMFR (95% CI)
**0**	33	5.4 (3.4 - 8.7)	5.2 (3.3 - 8.1)	3.6 (2.3 - 5.6)	2.7 (1.7 - 4.3)
**1**	25	8.5 (4.6 - 15.5)	6 (3.9 - 9.3)	7 (4.1 - 11.8)	6.1 (3.9 - 9.8)
**2**	17	4.5 (2.4 - 8.7)	6.6 (3.4 - 12.8)	3.7 (1.9 - 7.3)	3.4 (1.9 - 6)
**3**	21	4.4 (2.2 - 8.5)	3.4 (1.7 - 6.6)	2.5 (1.4 - 4.3)	2.3 (1.6 - 3.3)
**4**	21	2.1 (1.3 - 3.5)	2.4 (1.7 - 3.5)	2 (1.3 - 3)	1.8 (1.2 - 2.5)
**5**	24	4.6 (3 - 7.1)	3.8 (2.2 - 6.6)	2.7 (1.9 - 3.9)	2.4 (1.7 - 3.4)
**6**	14	5.4 (3.4 - 8.7)	4.5 (1.7 - 11.8)	1.9 (1.4 - 2.6)	2.8 (1.7 - 4.4)
**7**	10	8.6 (3.6 - 20.2)	5.7 (2.5 - 12.8)	2 (1.4 - 2.8)	2.5 (1.3 - 4.6)
**8**	10	11.3 (6 - 21.2)	2.3 (1.1 - 4.6)	2.8 (1.5 - 5.3)	3.7 (1.7 - 8.2)
**9**	18	16 (7.5 - 34.1)	7.4 (4.1 - 13.4)	2.8 (1.7 - 4.8)	4.7 (2.4 - 9)
**10**	13	5.8 (3.5 - 9.6)	2.5 (1.4 - 4.5)	2.2 (1.3 - 3.8)	3.2 (1.7 - 6.3)
**11**	12	12 (6 - 23.9)	5 (2.2 - 11.5)	2.8 (1.3 - 6.2)	2.7 (1.4 - 5.2)
**12**	12	9 (4.5 - 18.1)	6.3 (3.3 - 12.3)	2.2 (1.3 - 3.8)	4.5 (2.2 - 9)
**13**	7	11.9 (3.7 - 38)	5.4 (1 - 29.2)	2.4 (0.7 - 8.7)	4 (1.3 - 12.1)
**14**	8	10.4 (3.1 - 34.3)	5.7 (2.8 - 11.3)	2.8 (1.1 - 7.2)	1.8 (1.1 - 3)
**15**	9	8.6 (2.5 - 29.7)	6.9 (1.9 - 25.1)	2.3 (1.3 - 4.2)	5.4 (1.8 - 16.4)
**16**	20	13.9 (7.6 - 25.6)	6.7 (4.1 - 11.1)	3.6 (2.3 - 5.7)	4.6 (2.7 - 7.9)
**17**	9	5 (1.4 - 18.6)	8.6 (2.7 - 28)	2.5 (0.9 - 7.1)	2.2 (1.2 - 3.8)
**18**	13	15.2 (7.7 - 29.7)	3.2 (1.4 - 7.6)	3.1 (1.6 - 5.9)	3.8 (2 - 7.3)
**19**	14	9.3 (3.3 - 26.3)	5.7 (2.2 - 14.5)	3 (1.4 - 6.5)	3.1 (1.2 - 7.9)
**20**	8	14.7 (5.4 - 39.9)	2.8 (1.2 - 6.4)	3.7 (1.5 - 9)	3.7 (1.3 - 10)
**21**	3	16 (0.8 - 315.8)	5 (0.7 - 36.8)	4 (0.2 - 78.9)	5 (0.7 - 36.8)

GMFR = geometric mean fold rise; CI = confidential interval.

Supplementary figure S1. Flow cytometry analysis of T cell activation, proliferation, and apoptosis.

T cells and B cells from healthy donors (n = 4) were stimulated with anti-CD3/CD28 beads and CD40L, respectively, in the presence or absence of 2 µM MTX. (A) Representative plots illustrating the sequential gating strategy to identify T cell populations. Cells were gated for morphological lymphocytes, singlets, and live cells. $CD4^+$ T cells were subdivided into naive T cells (TN; $CD45RA^+CCR7^+$), central memory T cells (TCM; $CD45RA^-CCR7^+$), and effector memory T cells (TEM; $CD45RA^-CCR7^-$). TCM and TEM populations were analyzed collectively as the memory T cells (TM) for all readouts. (B) Representative plots of gating strategy to identify B cell populations. $CD19^+$ B cells were subdivided into naive B cells (BN; IgD^+CD27^-), memory B cells (BM; IgD^-CD27^+), and antibody-secreting cells (ASC; $IgD^-CD27^{hi}CD38^{hi}$). (C) Representative histograms of CD25 expression on CD4 T cells at 48 and 96 hours. (D) Representative plots of EdU/FxCycle based cell cycle assay on CD4 T cells at 48 hours. (E) Representative histograms of CellTrace based proliferation assay on CD4 T cells. (F) Representative plots of Annexin V based apoptosis assay on CD4 T cells at 96 hours.

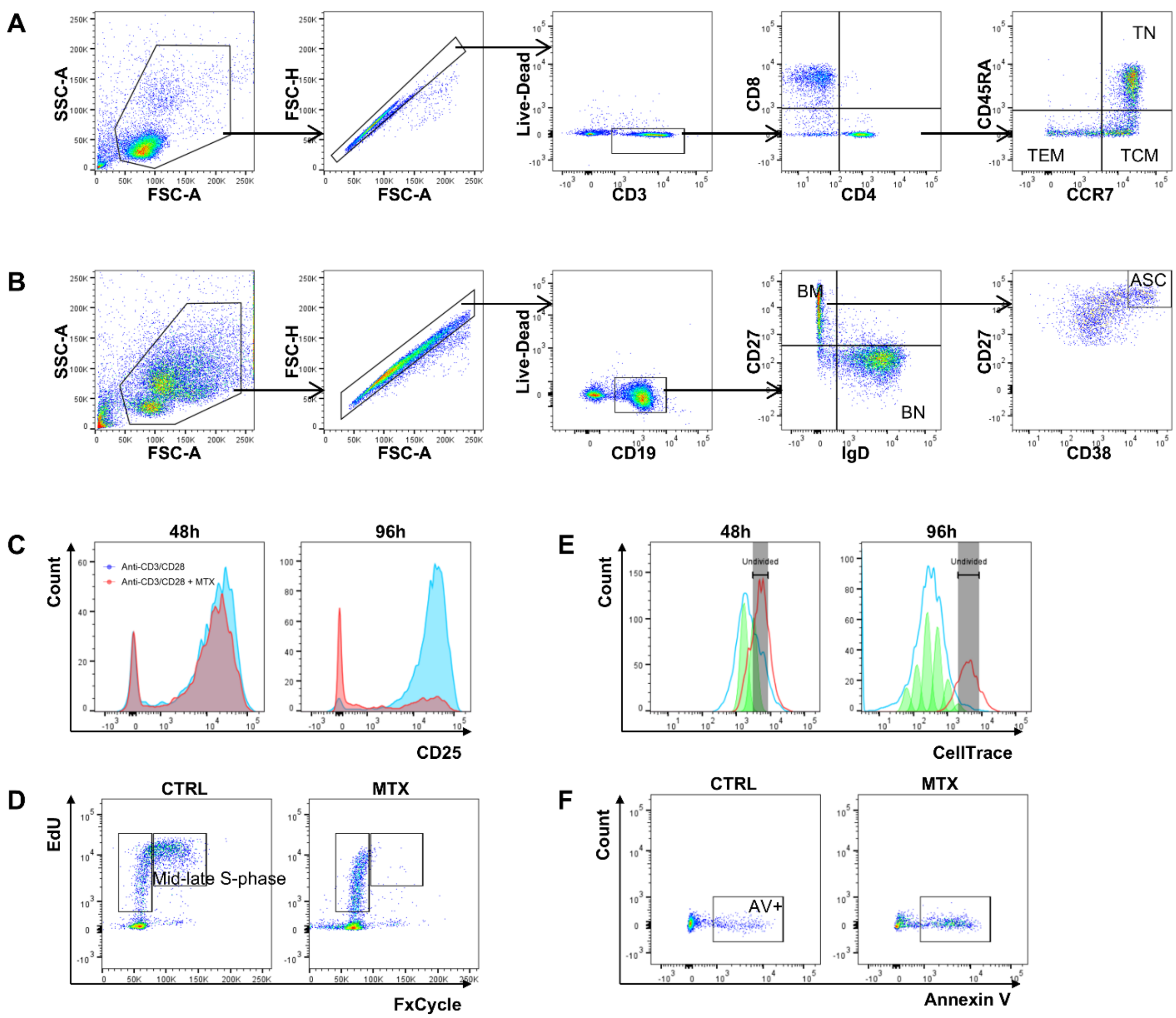

Supplementary figure S2. CD38 expression on T cells.

(A) $CD4^+$ T cells from healthy donors (n = 4) were stimulated with anti-CD3/CD28 beads in the presence or absence of 2 µM MTX and analyzed by flow cytometry. Schematic of the experimental design and mean fluorescence intensity (MFI) of CD38 on naïve (TN) and memory (TM) T cells are shown. (B) $CD4^+$ T cells from healthy donors were stimulated with anti-CD3/CD28 beads and treated with a 24-hour pulse of MTX (2 µM) at defined activation time points. Schematic of the experimental design and MFI of CD38 on TN and TM cells are shown. Data are presented as mean ± SD. Statistical significance: *P < 0.05, **P < 0.01, ***P < 0.001, ****P < 0.0001. Multiple comparisons were corrected using the Bonferroni method.

A

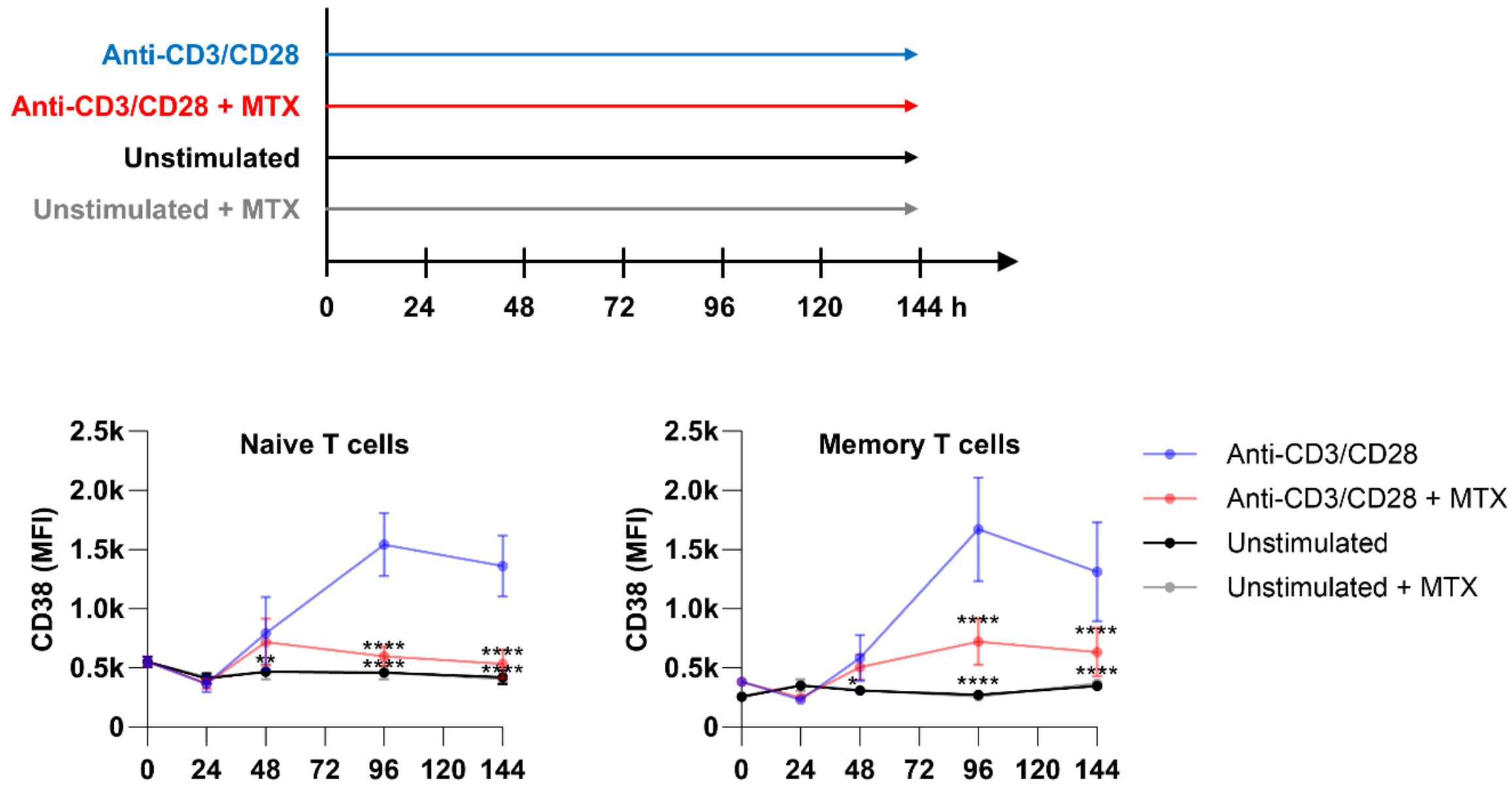

B

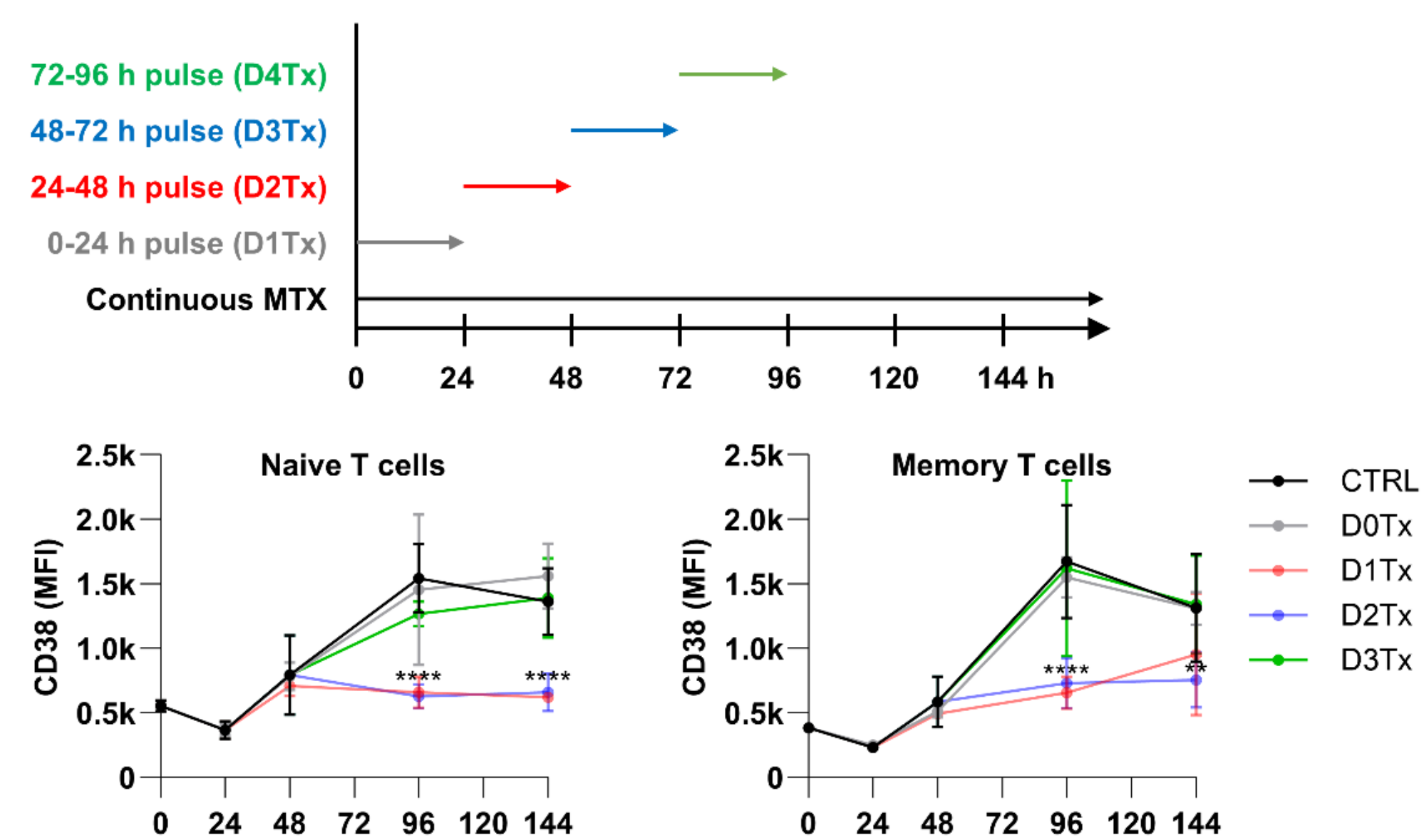

Supplementary figure S3. Effects of methotrexate on antibody production.

B cells were stimulated in the presence of MTX (2 µM) either continuously (A) or as a 24-hour pulse (B), and the number of antibody-secreting cells (ASCs) and total Ig production were quantified on Day 6 of stimulation.

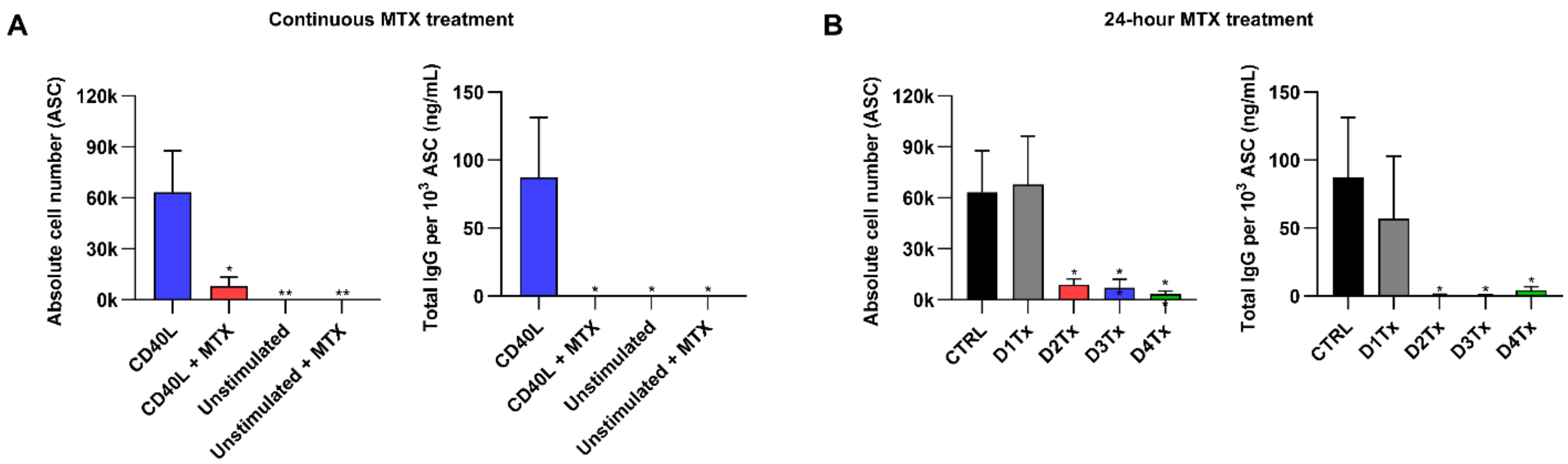

Supplementary figure S4. Expression of MTX transporters and adenosine-regulating enzymes in activated T cells.

(A) Relative mRNA expression of ABC transporters (ABCB1 and ABCC1) in $CD4^{+}$ T cells over time following stimulation. (B) Expression of the ectoenzymes CD39 and CD73 in stimulated T cells.

A

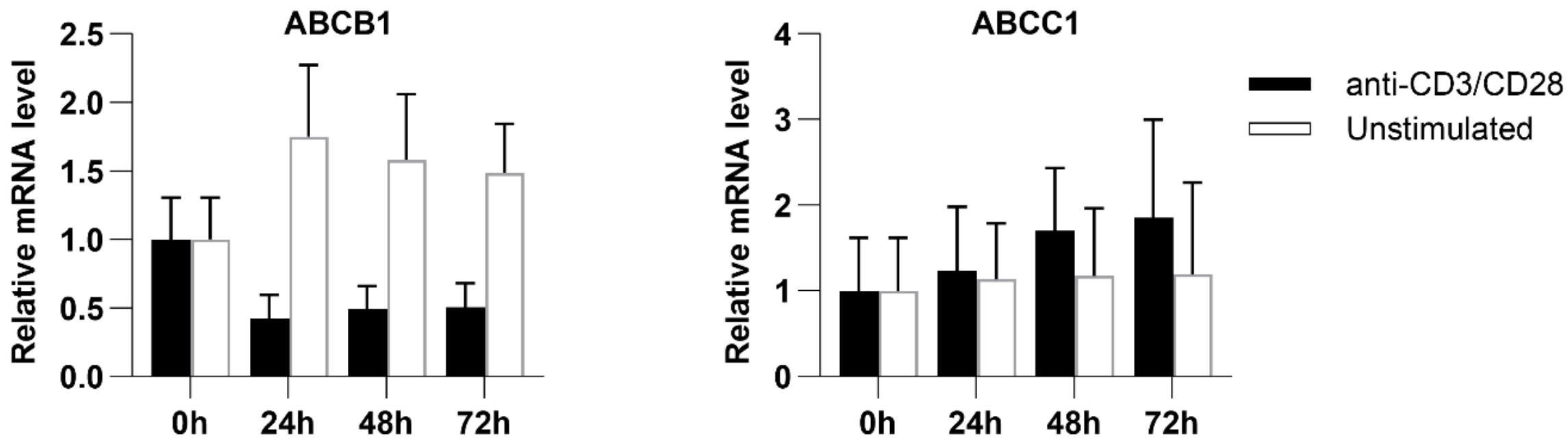

B

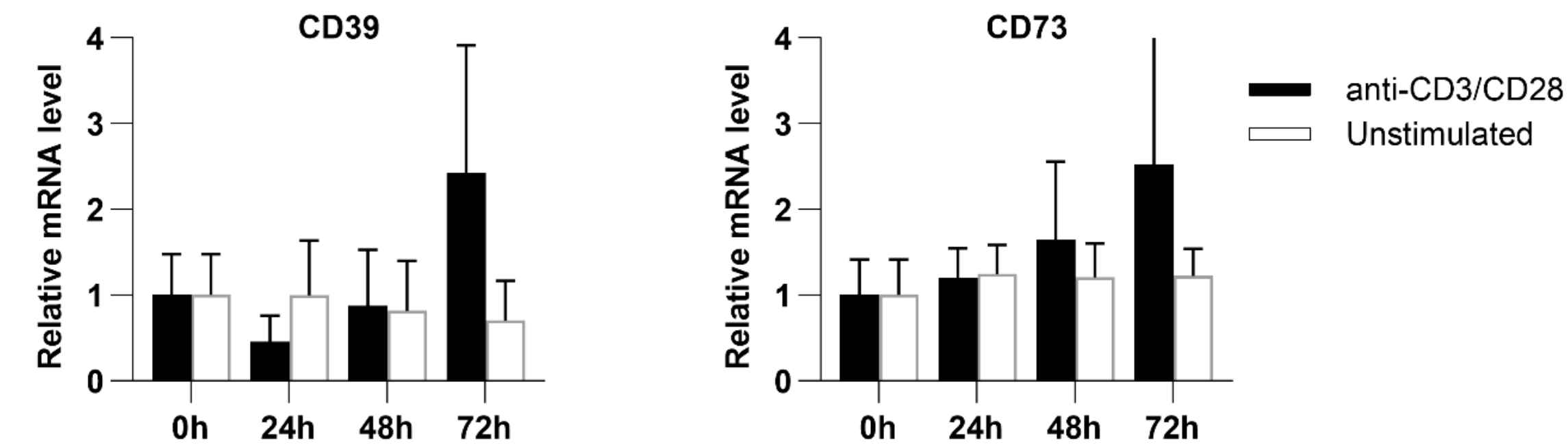

Supplement: Supplementary file 1 [file DataSheet1.pdf]
